# When acute myocardial infarction meets renal abscess: Case report and literature review

**DOI:** 10.1097/MD.0000000000040655

**Published:** 2024-11-22

**Authors:** Xiyan Zhu, Hongyun Shu, Sisi Han, Jianhong Li, Haicui Su, Qiaowen Li

**Affiliations:** a Department of Cardiovascular Medicine, The Affiliated Qingyuan Hospital (Qingyuan People’s Hospital), Guangzhou Medical University, Guangdong, China; b Institute of Gerontology, Guangzhou Geriatric Hospital, Guangzhou Medical University, Guangzhou, China.

**Keywords:** acute myocardial infarction, renal abscess, treatment

## Abstract

**Rationale:**

Acute myocardial infarction (AMI) is the leading global cause of death from cardiovascular disease, and the mortality rate increases in the presence of comorbidities such as renal abscess. The treatment of AMI combined with renal abscess is challenging, especially in combination with urinary tract obstruction, as percutaneous coronary intervention (PCI) can lead to progression of the renal abscess and deterioration of renal function. Currently, there is no consensus on the treatment of renal abscess in AMI.

**Patient concerns:**

We reported a case of a 74-year-old male patient with acute non-ST-segment elevation myocardial infarction combined with urinary tract obstruction. During his hospitalization, the hydronephrosis progressed to a renal abscess, which we punctured and drained. He ultimately underwent twice PCI and surgical relief of the ureteral obstruction shortly thereafter.

**Diagnoses:**

He was diagnosed with acute non-ST elevation myocardial infarction on admission, unfortunately, his hydronephrosis progressed into a renal abscess after the first PCI, which made further treatment difficult.

**Interventions:**

We performed 2 coronary angiography examinations and implanted a stent in the stenotic coronary artery during the second procedure, which was preceded by an aggressive regimen of antibiotics and puncture and drainage of the renal abscess, which set the stage for the second PCI.

**Outcomes:**

We successfully performed coronary revascularisation to treat his coronary artery disease. One month later, his renal abscess drain was removed and the ureteral obstruction was finally resolved after undergoing percutaneous nephrolithotripsy for ureteral stone extraction.

**Lessons:**

The occurrence of renal abscesses is rare and may be unavoidable in those patients with preexisting structural lesions in the urinary tract where coronary angiography will increase the incidence of renal abscesses. Aggressive anti-infective therapy and drainage of pus by puncture will help the renal abscess to heal, and repeat coronary angiography has been shown to be safe in the meantime.

## 1. Introduction

Acute myocardial infarction (AMI) is a disease with a very high mortality rate. In clinical diagnosis and treatment, we assess and grade the patients according to their overall condition and treated the diseased vessel with percutaneous coronary intervention (PCI) for revascularisation as early as possible after exclusion of contraindications.^[1,2]^ The timely and effective intervention can greatly reduce the mortality rate and improve the prognosis. Obstructive hydronephrosis caused by urinary stones is a common disease in the urinary tract and a high-risk factor for the development of renal abscess. Inadequate treatment of renal abscess induces sepsis, septicemia, infectious shock, and even death.^[3,4]^ Whether the evolution of AMI, the diagnostic and treatment process, and the occurrence, development, and outcome of renal abscess are correlated has not been studied in detail. Here, we review the mechanisms involved in the development of renal abscesses and discuss how we can rationalize treatment when renal abscesses meet AMI.

## 2. Case description

**Figure 1.**
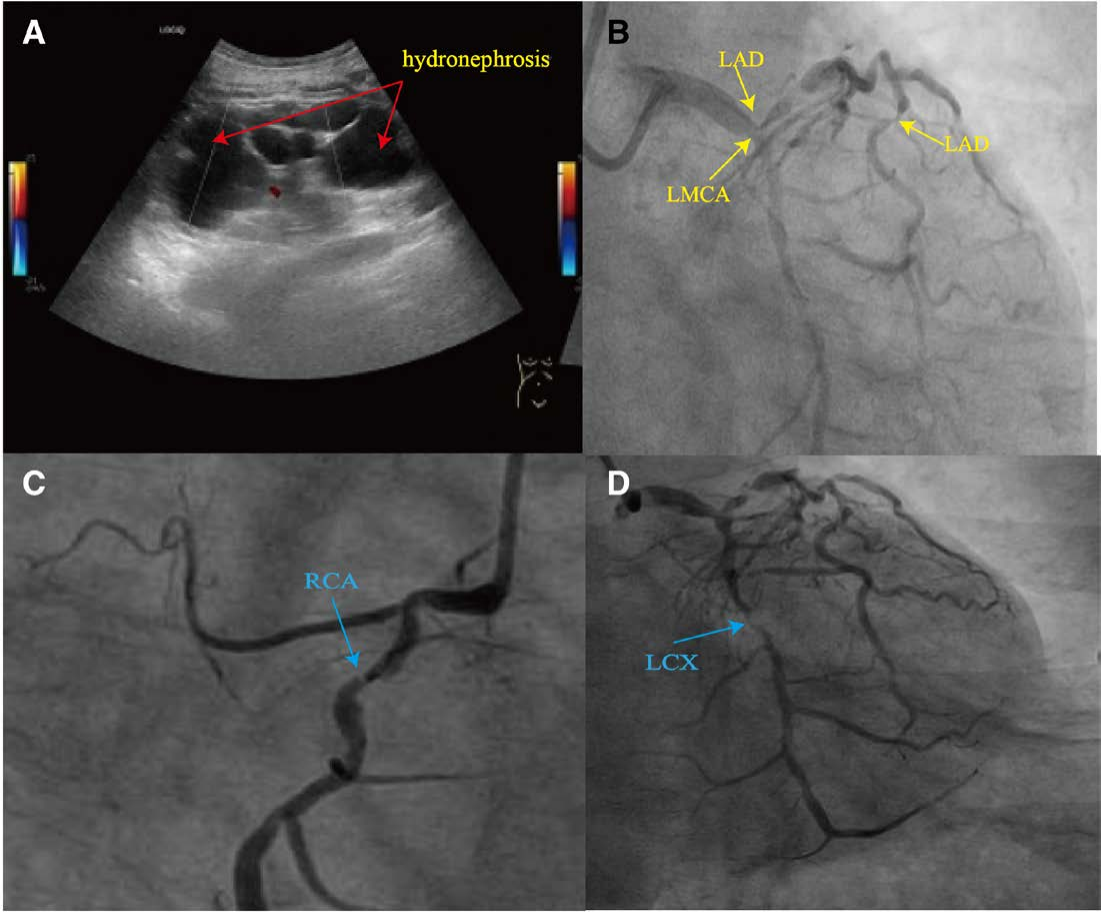
(A) Urological ultrasound at presentation: the renal pelvis and calyces of the left kidney were dilated with thin parenchyma, the collecting system was dilated with a width of about 34 mm, and several strong echoes were seen in the calyces, the larger of which was about 13 × 4 mm in size, accompanied by acoustic shadows. A strong echo of about 17 × 7 mm with acoustic shadow was detected in the upper part of the left ureter, and the ureter above it was dilated. (B) The first CAG examinations: there was 60% stenosis of the LMCA, 90%–95% stenosis of the LAD artery. (C) 90% proximal stenosis of the right coronary artery. (D) 95% stenosis of the LCX. CAG = coronary angiography, LAD = left anterior descending, LCX = left circumflex artery, LMCA = left main coronary artery.

**Figure 2.**
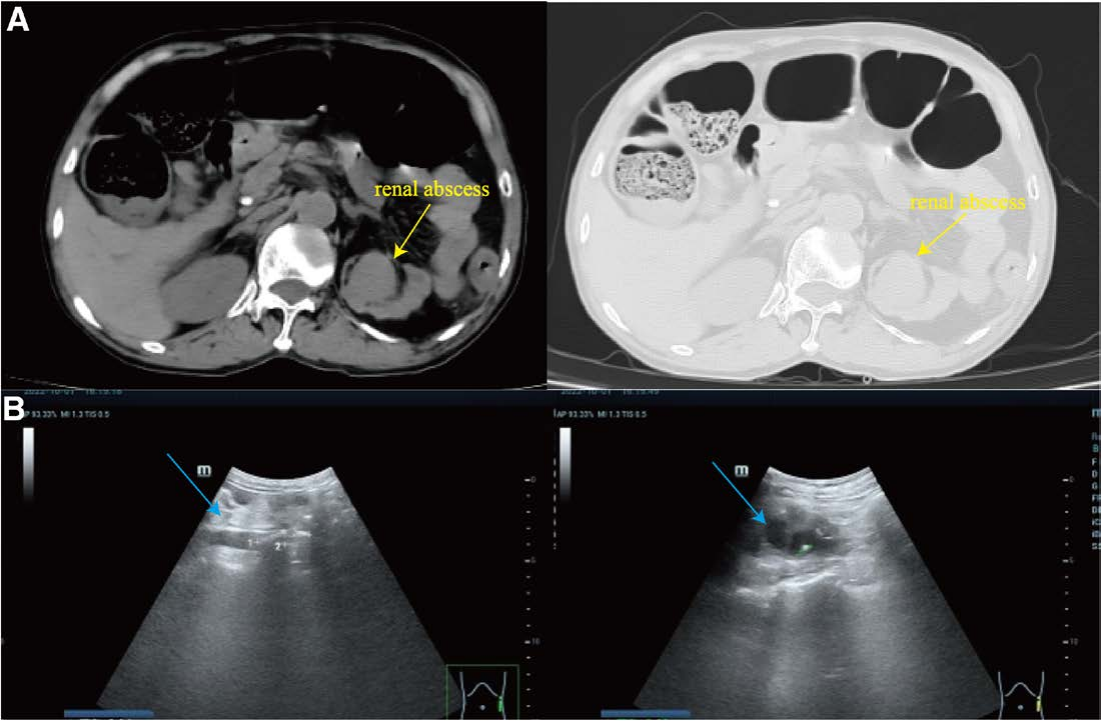
(A) Abdominal CT (the 2nd day after surgery): nodular hyperdense shadow with a diameter of approximately 17 mm × 8 mm × 7 mm was seen in the upper part of the left ureter, above which fluid is accumulated in the left ureter and the left kidney, with a CT value of about 38 HU, quasi-circular low-density foci was seen in the left kidney parenchyma, with a diameter of about 38 mm and a CT value of about 2 HU. (B) Urological ultrasound (4 mo after discharge): the left kidney is still normal in size, the parenchyma is thin, and the internal structure is still clear. CT = computed tomography.

A 74-year-old Chinese male with a history of hypertension for more than 8 years presented to the emergency room with shortness of breath and palpitations for more than 10 days. His electrocardiogram (ECG) did not show ST-segment elevation, however, laboratory tests were notable for a cardiac troponin I (cTnI) of 19.11 µg/L (reference range: 0–0.014 µg/L), myoglobin of 29.86 µg/L (reference range: 10–80 µg/L), furthermore, C-reactive protein (CRP) levels of 54.75 mg/L(reference range: 0–10 mg/L), the white blood cell (WBC), neutrophil counts and creatinine levels were normal. Initially, he was diagnosed with acute non-ST-segment elevation myocardial infarction. We assessed the patient using the GRACE risk scale, and the patient was in the high-risk group and had evidence of early (within 24 hours) PCI. However, the patient had severe heart failure combined with infection and we postponed PCI treatment due to critical condition and inability to tolerate surgery. He was commenced on antithrombotic (aspirin and ticagrelor) and secondary preventative therapies before undergoing invasive coronary angiography (CAG), in addition to this, due to the patient’s current state of infection, we prophylactically administered broad-spectrum antibiotics(cefuroxime). On admission, urological ultrasound showed left upper ureteral stone with heavy left hydronephrosis (Fig. 1A) and multiple left kidney stones. The patient’s urine output and creatinine level were normal, so we did not further manage the renal stones and hydronephrosis. We performed the first CAG examinations on the sixth day after admission. The CAG showed that multiple coronary stenoses (Fig. 1B and D). However, the patient refused to implant a stent for revascularisation so we lost the optimal timing of the procedure. After recommunicating with the patient, on the 10th day after admission, we performed CAG again via the right femoral artery access and completed PCI and stenting in the left main coronary artery, right coronary artery, and the left circumflex artery. On the 2nd day after surgery (11th day after admission), the patient suffered abdominal pain, and the examination was positive for right lower abdominal pressure with muscle tension and no rebound pain. The patient was immediately taken for a computed tomography (CT) and the abdominal CT scan (Fig. 2A) suggested a stone in the upper left ureter, with fluid and pus accumulation in the left ureter and left kidney above the stone. His WBC count was 37.8 × 10^9^/L and neutrophil count was 35.44 × 10^9^/L, procalcitonin was 26.32 ng/mL (reference range: <0.5 ng/L), which suggested that the patient currently had a severe systemic infection, so we adjusted the anti-infective regimen (using imipenem instead of cefuroxime). In addition, the patient’s rising creatinine level (126.5 µmol/L) suggested that his renal function was deteriorating, so we performed ultrasound-guided renal abscess puncture and drainage. Finally, after 11 days of treatment, the creatinine, WBC, and neutrophil counts normalized, and the patient was discharged from the hospital after the left nephrostomy drain was left in place. At the follow-up visit 4 months later, his urologic ultrasound had been almost normal (Fig. 2B).

## 3. Discussion

We reported the case of a patient with AMI combined with renal abscess, in which the patient’s renal function was normal at the initial stage of the disease, and the urologic ultrasound only suggested hydronephrosis. However, during our intervention, this patient developed a renal abscess and impaired renal function, suggesting that the initial CAG examination promoted renal abscess formation.

Obstructive effusion due to urinary stones and the ensuing infection are considered to be the most important primary risk factors for renal abscesses, furthermore, ureteral stenosis, necrosis of the renal papilla, obstruction of the renal pelvic-ureteral junction, and malignant tumors can also lead to renal abscesses.^[5]^ Hydronephrosis caused by urinary tract obstruction leads to decreased peristalsis of the ureter, which accelerates bacterial colonization. The purulent urine in the renal pelvis can seep into the renal parenchyma and destroy it due to infection, eventually leading to renal abscess.^[6]^ In this process, the pathophysiologic results of AMI will accelerate the progression of renal abscess. As we know, the occurrence of AMI can lead to a decrease in ejection capacity, causing systemic hypoperfusion, resulting in multiple organ ischemia and hypoxia, in which the kidneys are very sensitive to changes in peripheral blood volume, and prerenal insufficiency can lead to impaired renal function. Moreover, a variety of inflammatory factors are released into the circulation during AMI,^[7]^ which can exacerbate ischemia-reperfusion injury. The contrast agents used in interventional therapy, which increase blood viscosity, osmotic pressure, and cytotoxicity, can lead to renal medullary injury.^[8]^

Renal abscess causes the expansion of renal pelvis and renal calyces, which further compresses the renal medulla, leading to increased pressure of blood circulation in the medulla, making the kidneys ischemic, and at the same time, urine can not be discharged, toxins accumulate in the body, and persistent urinary obstruction leads to aggravation of renal pus and formation of a vicious cycle of damage to renal function.

Acute-onset renal abscess is the most common clinical type, which is characterized by acute onset of fever, chills, vomiting, flank pain, and general weakness.^[9]^ Studies have demonstrated that moderate to severe hydronephrosis, a history of fever, decreased serum albumin, and elevated CRP, procalcitonin, leukocytes, and neutrophils are risk factors for the progression of urinary tract obstructive hydronephrosis to renal abscess.^[10–12]^ The treatment of obstructive nephrolithiasis includes surgery and drugs, and the surgical aspects include double J-tube implantation, percutaneous nephrolithotomy, extracorporeal shock wave lithotripsy, and percutaneous nephrolithotripsy for stone removal. Percutaneous drainage using ultrasound and/or CT guidance has been considered an effective tool for intraabdominal abscesses.^[13,14]^

Urinalysis and urine bacterial culture results cannot be used as the basis for the diagnosis of renal abscess, because some patients with obstructive renal abscess have a long course of the disease, and the affected side of the ureter is completely occluded, which leads to the above tests are likely to be negative results. As the patient we reported, multiple urine cultures during hospitalization were negative. Therefore, it may be more effective to apply antibiotics empirically and adjust the type of antibiotic according to the response to treatment. Therefore, it may be more effective to apply antibiotics empirically and adjust the type of antibiotics according to the response to treatment. According to our knowledge, the most common pathogens of urinary tract stones and hydronephrosis that cause concurrent acute infections in the urinary tract are gram-negative bacteria.^[15,16]^ Escherichia coli is predominant, and anti-infective treatment with ceftriaxone or ampicillin/sulbactam is often recommended clinically.^[17]^ Intrarenal abscesses without concomitant urinary obstruction are usually successfully treated with antibiotic therapy alone.^[18]^

In summary, when a patient with AMI is already at risk for urinary tract obstruction, it is important to open the diseased vessel in a timely manner to preserve cardiac function as much as possible. This ensures a more normal blood perfusion to the kidneys and slows down the deterioration of renal function. Aggressive management of stones and treatment with different surgical modalities depending on the stone condition and targeted interventions are important. Percutaneous drainage in conjunction with antibiotics is an effective initial therapeutic modality for renal abscess.^[14]^ However, during the course of this patient’s consultation, we neglected to manage the ureteral obstruction situation, which led to a severe renal abscess presentation and severe infection after the second PCI. We hypothesized that the patient’s renal abscess had already begun after the first CAG and that early release of the ureteral obstruction might have prevented further development of the renal abscess.

## 4. Conclusion

Renal function should be attended to during AMI treatment, cardiac function should be preserved as much as possible, and multiple imaging over a short period of time should be avoided when risk factors for urinary tract obstruction have been combined, and appropriate hydration or pharmacological interventions will reduce renal damage. Drainage of the abscess, in addition to early antibiotic therapy, is effective in resolving renal abscesses caused by obstruction.

## Author contributions

**Writing—original draft:** Hongyun Shu, Xiyan Zhu.

**Supervision:** Qiaowen Li.

**Writing—review & editing:** Jianhong Li, Sisi Han, Haicui Su.

## References

[1] Bhatt DL. Percutaneous coronary intervention in 2018. JAMA. 2018;319:2127–8. 29800163 10.1001/jama.2018.5281

[2] Collet JP Thiele H Barbato E. ESC Scientific Document Group. 2020 ESC Guidelines for the management of acute coronary syndromes in patients presenting without persistent ST-segment elevation. Eur Heart J. 2021;42:1289–367. 32860058 10.1093/eurheartj/ehaa575

[3] Zhu Z Cui Y Zeng H. The evaluation of early predictive factors for urosepsis in patients with negative preoperative urine culture following mini-percutaneous nephrolithotomy. World J Urol. 2020;38:2629–36. 31828354 10.1007/s00345-019-03050-9

[4] Kam J Yuminaga Y Beattie K. Single use versus reusable digital flexible ureteroscopes: a prospective comparative study. Int J Urol. 2019;26:999–1005. 31448473 10.1111/iju.14091

[5] Ng CK Yip SK Sim LS. Outcome of percutaneous nephrostomy for the management of pyonephrosis. Asian J Surg. 2002;25:215–9. 12376218 10.1016/S1015-9584(09)60178-0

[6] Maiti A Saha D Das A. Emphysematous pyelitis: an entity distinct from emphysematous pyelonephritis. Am J Med Sci. 2017;353:505. 28502342 10.1016/j.amjms.2016.07.005

[7] Prabhu SD Frangogiannis NG. The biological basis for cardiac repair after myocardial infarction: from inflammation to fibrosis. Circ Res. 2016;119:91–112. 27340270 10.1161/CIRCRESAHA.116.303577 PMC4922528

[8] Zhang F Lu Z Wang F. Advances in the pathogenesis and prevention of contrast-induced nephropathy. Life Sci. 2020;259:118379. 32890604 10.1016/j.lfs.2020.118379

[9] Jaik NP Sajuitha K Mathew M. Renal abscess. J Assoc Physicians India. 2006;54:241–3. 16800353

[10] Desai M De Lisa A Turna B. The clinical research office of the endourological society percutaneous nephrolithotomy global study: staghorn versus nonstaghorn stones. J Endourol. 2011;25:1263–8. 21774666 10.1089/end.2011.0055

[11] Weinberg AE Patel CJ Chertow GM Leppert JT. Diabetic severity and risk of kidney stone disease. Eur Urol. 2014;65:242–7. 23523538 10.1016/j.eururo.2013.03.026 PMC3866968

[12] Kabeya Y Kato K Tomita M. Associations of insulin resistance and glycemic control with the risk of kidney stones. Intern Med. 2012;51:699–705. 22466823 10.2169/internalmedicine.51.6426

[13] Yen DH Hu SC Tsai J. Renal abscess: early diagnosis and treatment. Am J Emerg Med. 1999;17:192–7. 10102326 10.1016/s0735-6757(99)90060-8

[14] Tu MQ Li JH Fu XC. [Clinical analysis of 28 cases of calculous pyonephrosis undergoing B-ultrasound-guided renal puncture and drainage followed by secondary percutaneous nephrolithotomy]. Zhonghua Yi Xue Za Zhi. 2019;99:3005–7. 31607033 10.3760/cma.j.issn.0376-2491.2019.38.008

[15] Landgren M Odén H Kühn I Osterlund A Kahlmeter G. Diversity among 2481 *Escherichia coli* from women with community-acquired lower urinary tract infections in 17 countries. J Antimicrob Chemother. 2005;55:928–37. 15886265 10.1093/jac/dki122

[16] Malani AN Kauffman C. A Candida urinary tract infections: treatment options. Expert Rev Anti Infect Ther. 2007;5:277–84. 17402842 10.1586/14787210.5.2.277

[17] Florido C Herren JL Pandhi MB Niemeyer MM. Emergent percutaneous nephrostomy for pyonephrosis: a primer for the on-call interventional radiologist. Semin Intervent Radiol. 2020;37:74–84. 32139973 10.1055/s-0039-3401842 PMC7056339

[18] Dembry LM Andriole VT. Renal and perirenal abscesses. Infect Dis Clin North Am. 1997;11:663–80. 9378929 10.1016/s0891-5520(05)70379-2

